# Healthcare Professional Training Regarding Gestational Weight Gain: Recommendations and Future Directions

**DOI:** 10.1007/s13679-021-00429-x

**Published:** 2021-02-20

**Authors:** Ellinor K. Olander, Briony Hill, Helen Skouteris

**Affiliations:** 1 Centre for Maternal and Child Health Research, School of Health Sciences, City, University of London, Northampton Square, London, EC1V 0HB UK; 2 National Health and Medical Research Council Early Career Fellow, Monash Centre for Health Research and Implementation, Monash University, Level 1, 43-51 Kanooka Grove, Clayton, VIC 3169 Australia; 3 Health and Social Care Improvement and Implementation Science, Monash Centre for Health Research and Implementation, School of Public Health and Preventive Medicine, Monash University, Level 1, 43-51 Kanooka Grove, Clayton, VIC 3169 Australia; 4 Warwick Business School, Warwick University, Coventry, CV47AL UK

**Keywords:** Pregnancy, Weight gain, Midwife, Obstetrician, Training, Education

## Abstract

**Purpose of review:**

The aim of this review was to summarise recent evaluations of healthcare professional training regarding gestational weight gain and provide recommendations for future training.

**Recent findings:**

A number of evaluated healthcare professional training sessions regarding gestational weight gain show promising results in terms of increased participant confidence and knowledge and impact on women’s outcomes. It is clear that the interventions which have also implemented resources in the practice environment to support training are the ones most likely to influence gestational weight gain.

**Summary:**

Support from healthcare professionals are key to influence pregnant women’s weight gain and should be offered within the standard curriculum and through mandatory training. Factors influencing this support include women’s and healthcare professional characteristics, interpersonal and healthcare system and policy factors. All of these need to be considered when developing healthcare professional training to support women with their gestational weight gain.

## Introduction

Excessive gestational weight gain is a modifiable risk factor associated with risks for both mother and baby. For example, excessive gestational weight gain is associated with large for gestational age babies and caesarean delivery [1]. It is also associated with long-term weight retention [2] and women starting a subsequent pregnancy with a higher body mass index (BMI). Research has shown that women in all BMI categories gain more than what is recommended by the Institute of Medicine’s gestational weight gain guidelines [1, 3]. For example, in one study, almost 50% of women gained more weight than recommended [4]. As such, excessive gestational weight gain has been seen as the fuel for future obesity in both mother and baby. Worryingly, around 20% of women gain an inadequate amount of weight, which is associated with risks such as small for gestational age and pre-term birth [1]. Hence, only around one-third of women gain within current recommendations, explicating the importance of targeting this clinical issue.

Study findings show consistently that healthcare professionals such as obstetricians, midwives and family physicians/general practitioners do not feel confident or knowledgeable enough to provide support regarding gestational weight gain [5–7]. A recent systematic review found that healthcare professionals lack knowledge regarding current gestational weight gain guidelines and that training is needed urgently [8]. Other factors preventing healthcare professionals from discussing gestational weight gain include lack of time and resources such as services they can refer to [7], not prioritising the issue [7] and concern that they may stigmatise women [7, 9–12]. A further concern is the effectiveness of the gestational weight gain conversations, reported to be infrequent or inaccurate [12, 13]. Supporting this are the findings that there is little association between advice given to women and their weight gain [14, 15].

Without a confident and prepared workforce discussing weight gain with pregnant women, it is not surprising that women often report not being aware of gestational weight gain guidelines [16] or the risks associated with excessive and inadequate gestational weight gain [5]. Importantly, pregnant women expect their maternity care provider to inform them of everything significant in pregnancy, so without a discussion about gestational weight gain, women may not think their weight gain is important [5]. This may subsequently lead to women declining weight management support [17, 18]. Alternatively, women may seek information elsewhere such as the internet or from family and friends [19] which may not be accurate or evidence-based information. Receiving advice from family and friends has recently been found to be associated with gaining outside the recommended weight ranges [20].

It is therefore clear that healthcare professionals need training and support to discuss weight gain with pregnant women [3, 6, 7, 13, 21, 22••]. This review starts with a brief summary of the risks associated with excess weight gain in pregnancy and what women may be at higher risk of this weight gain. It continues with the main aim of this review—to summarise evaluations of healthcare professional training regarding gestational weight gain to date and provide suggestions for promising strategies to facilitate future training development and delivery. Recommendations for how healthcare professionals can be supported to implement effective weight management conversations within routine antenatal care are also provided ahead of a discussion of future directions.

## Pathophysiology and Risks Associated with Excess Weight Gain in Pregnancy

Pregnant women go through dramatic physiological changes to support foetal growth, including changes to the cardiovascular, endocrine, vascular and metabolic systems (for a detailed summary, see reference 23). Gestational weight gain includes water, fat, protein and minerals deposited in the placenta, foetus, uterus, amniotic fluid, mammary gland, blood and adipose tissue [23, 24]. These components make women’s gestational weight gain vary greatly, with mean total gestational weight gain for a woman with a pre-pregnancy BMI in the healthy category ranging from 10.0 to 16.7 kg [23]. The mean weight is higher for younger women such as adolescents and those women carrying twins or triplets [23]. It is therefore vital that women receive weight gain support that is tailored to their situation and pregnancy [24].

There are both short- and long-term risks for the mother and baby associated with excess weight gain. These short-term risks include gestational hypertension, pre-eclampsia [24], gestational diabetes [24, 25] and large-for-gestational age babies [1, 24]. There is also a higher risk for intervention during birth and caesarean delivery [24, 26], associated with longer hospital stay and increased healthcare costs. Longer term risks includes future overweight and obesity [2, 24], potentially affecting a future pregnancy. Excessive weight gain is also associated with obesity in the child in both the short and long term [27, 28].

## Who May Gain an Excessive Amount of Weight in Pregnancy?

To help tailor training, it is important to examine epidemiological evidence on who may gain excessive weight during pregnancy. There is consistent evidence that women living with pre-pregnancy overweight or obesity gain an excessive weight in pregnancy [29]. Living in a deprived community or on low income is another predictor of excessive weight gain [24]. Other demographic factors such as age are also important, with younger women gaining more weight than older women [30]. It is unclear if parity is associated with excessive gestational weight gain [31].

There are also behavioural factors to consider. Physical activity [29, 32, 33] and a healthy diet [33, 34] seem to protect against excessive gestational weight gain. On the other hand, high total energy intake and high intake of fried food and dairy are associated with higher gestational weight gain [32]. Finally, psychosocial factors such as negative body image [35, 36], low self-efficacy [34] and inaccurate perceptions regarding own weight [36] are associated with increased gestational weight gain.

## Healthcare Professional Training Regarding Gestational Weight Gain

Training regarding gestational weight gain is rare [22••] with few healthcare professionals receiving training as part of their healthcare professional pre-registration education or as part of continuous professional development [9, 12, 37, 38]. Furthermore, training to date has been heterogeneous and varied in its content, delivery and methods of evaluation. Whilst focus has been on current practitioners such as obstetricians, midwives and family physicians/general practitioners, work is now also centring around the student population as the next generation of healthcare professionals.

### Current Practitioners

Kominiarek and colleagues, in their 2018 systematic review of studies that involved obstetric providers in interventions for either the perinatal management of obesity and/or gestational weight gain, included seven studies of which five focused on training regarding gestational weight gain [22••]. Whilst the included studies were too different to allow a meta-analysis, there were some common and promising findings. These included improved knowledge and confidence to discuss gestational weight gain after the training [39, 40] and adding resources to the practice environment such as automatic weight calculations and new documentation such as weight charts to record weight and associated guidelines [41, 42] to support discussion around gestational weight gain. Studies published since this review are summarised below.

Shwikar et al. 2020 conducted a pre-post study testing midwives knowledge of healthy eating and gestational weight gain guidelines after a 2-h training session, delivered via webinar or face-to-face [37]. Immediately, and then at 6-8 weeks after the training session, participants reported increased gestational weight gain knowledge. In a similar study, the knowledge of midwives and other healthcare professionals and their confidence regarding discussing gestational weight gain increased after four 30-min workshops delivered face-to-face or via online learning [43]. De Jersey and colleagues implemented gestational weight gain education into routine midwifery training, and after the 40-min session, participants reported increased confidence and knowledge regarding gestational weight gain [44].

Instead of examining healthcare professional knowledge or confidence, Weeks and colleagues examined pregnant women’s perceptions of how their healthcare professional delivered the 5A’s (Ask, Advise, Assist, Assess and Agree) in their antenatal appointment [45]. In their study, four obstetricians and 11 midwives received a 1-h workshop on the 5 A’s of gestational weight gain, a checklist and patient handout. Post-intervention, as compared to baseline, pregnant women scored higher for Ask (being asked about their ideas on how to manage their weight gain) and Advise (counselled on the exact recommended amount of weight to be gained throughout their pregnancy). Despite scores only increasing for Ask and Advice and not Assist, Assess and Agree, the authors concluded that the training may have clinical benefit.

In a similar study, a registered dietician attended two half days of training to learn how to provide healthy conversation skills to support healthy gestational weight gain [46]. The intervention group met with the trained dietician, and a control group met with a dietician who had not received the training. Through audio-recordings of conversations, a survey and focus groups with women and interview with the intervention dietician, it was shown that pregnant women in the intervention group felt better understood and supported and also spoke more during the sessions due to the dietician’s use of open questions. Whilst there was no difference in gestational weight gain between the two groups, there were positive behaviour changes regarding healthy eating and sedentary behaviour [47••].

In summary and taken together, research findings to date suggest that training can improve the confidence and knowledge of current healthcare professionals. The few studies that have examined women’s experiences after healthcare professional training report encouraging findings.

### Students

To prepare the healthcare workforce for future practice, it is important that medical and midwifery students are taught about gestational weight gain. It is therefore worrying that research suggests only minimal focus on gestational weight management advice is included in midwifery curricula [48] and that some students have to seek this information through extracurricular training [43]. One study found that only 12% of final year midwifery students knew the recommended weight gain range for a pregnant woman with a healthy BMI [49]. Including gestational weight gain training within the standard curriculum is important to ensure all students receive equitable education. In one study where obesity training was included in the curriculum, student doctors increased their confidence regarding counselling pregnant women regarding appropriate weight gain after the training [50]. Importantly, there were several aspects of this training that focused on the 5A’s, including wearing an empathy suit, reflection on their experience of this, and practice with patients and gestational weight gain was only a small part. More research is needed on what students learn as part of their standard curriculum, and this applies to all students studying for healthcare professions and who will care for pregnant women.

When training regarding gestational weight gain does take place, research suggests that this training should focus on creating positive attitudes towards providing weight management support and an environment where such interventions are promoted and reflect students own values [51]. Further suggestions include sensitive communication and awareness of not creating stigma [52]. Encouragingly, when student midwives have attended training regarding weight-related behaviour changes in pregnancy, they found this to be important and were able to reflect on and link it to clinical practice [53].

## Recommendations for Developing and Delivering Gestational Weight Gain Training

Our recommendations based on the current literature are divided into how to develop training, its content and how to deliver the training.

### Develop Training

A common concern amongst healthcare professionals is that discussing weight with women will make women feel stigmatised [11]. This concern is not unfounded, as it is a common belief amongst healthcare professionals that obesity can be prevented or cured with lifestyle change [54], leading to the perception that women should take full responsibility for their weight status and weight gain [55]. Facilitating the development of sensitive and appropriate training in gestational weight gain conversations requires meaningful stakeholder engagement [56]. Including women themselves is a critical component of training development and will help increase women’s satisfaction. Women can help co-produce and co-inform resources, referral forms and pathways to ensure these activities are woman centred.

### Training Content

When healthcare professionals are asked about what they include in their discussions regarding gestational weight gain, reports vary. However, most include weight gain targets, healthy eating and physical activity information and dispelling myths such as eating for two [6, 9]. This mirrors what is described in current training content, where focus has often been on *what* to say instead of *how* to say it. It is therefore encouraging to see that more recent research has focused on using the 5A’s to support gestational weight gain [45, 50] or healthy conversation skills [46] to provide healthcare professionals with tools on how to interact with women.

Recent training has also focused on contextualising weight gain and understanding the individual [50]. This includes the understanding that socio-economic position, culture, society and policies may also contribute to weight status and gain overall to reduce stigma against women [57]. This stigma has led to a phenomenon termed ‘mother blame’ where women are blamed for reproducing obesity across generations [55]. A recent review of interventions to assist healthcare professionals to improve the provision of lifestyle advice to patients (both in general and for women of reproductive age) identified a lack of stigma-reduction approaches [58], whilst there is also an underlying current of bias in communications about gestational weight gain from some obstetric providers [59]. For this review, only one study measured obesity stigma [50]. Thus, we need to improve our understanding of healthcare professionals’ implicit and explicit attitudes towards weight stigma, so these can be checked and targeted in education and training [57]. Furthermore, training in healthy conversation skills will facilitate an increase in the confidence of health professionals to have weight gain conversations with their pregnant patients [60].

Moreover, focusing on the *how* instead of the *what*, future-proofs training as guidelines are likely to change in line with new evidence emerging [6]. It is also important to consider the weight-related behaviours that influence gestational weight, healthy eating and physical activity, in training. This may include setting goals regarding behaviours [46] or use other behaviour change techniques [53]. Focusing on behaviours rather than weight may also avoid the concern that discussing weight will make women focus inappropriately and unhealthily on their weight [7].

There are inevitably several factors or circumstances that make delivering information and support regarding gestational weight gain more difficult. Some of these factors are summarised in Table 1 and include women’s characteristics, healthcare professional’s characteristics, interpersonal factors and healthcare system and policy factors. Regarding a woman’s characteristics, research suggests that healthcare professionals may feel more confident discussing gestational weight gain with older compared to younger woman and with women of a perceived higher compared to lower socio-economic status [21]. Regarding the characteristics of the healthcare professionals, their lack of self-efficacy initiating a non-stigmatising gestational weight gain conversation with women often prevents them from discussing weight gain [10, 13, 45]. Other factors that make discussions more difficult may be the presence of a woman’s partner or the woman not speaking the language of the country [61]. Further, one of the most commonly mentioned barriers is a lack of time [9, 19, 21, 45] which is part of healthcare system and policy factors. However, when healthcare professionals are provided with the opportunity of more time either as longer or additional appointments, discussing weight management becomes easier [62, 63]. It is important that training acknowledges and incorporates these factors. Whilst it is not always possible to provide healthcare professionals with more time, focus can instead be on how to discuss weight gain concisely and signpost to information and resources. Training also needs to acknowledge the diversity of pregnant women and incorporate resources for specific populations such as teenage women [44, 64], different cultures [37] and women at risk of eating disorders [65, 66].

**Table 1:** Examples of factors that may influence a healthcare professional’s weight gain discussion with a pregnant woman

Woman’s characteristics	Age [13, 21] Parity [13, 21] Socioeconomic status [13, 21] Pre-pregnancy BMI [12, 13, 21] Asking about healthy weight gain [9] Gestational weight gain to date [13] Culture, ethnicity and language [9, 47••]
Healthcare professional characteristics	Confidence, self-efficacy [13] Concerns about upsetting or stigmatising woman [10, 11, 13, 31] BMI of healthcare professional [9, 13]
Interpersonal factors	Established relationship with woman [3, 9, 21] Understood woman’s cultural background [21] Woman alone without partner [21, 47••] Assuming support is not useful/effective/not one’s role [13]
Healthcare system and policy factors	Time available [9, 19, 21, 31] Recording women’s weight [7, 13] Availability of weight management services [7, 10] When meeting woman, i.e. beginning or mid pregnancy [9, 10] Resources such as leaflets [9] Ability to refer patients to specialists (e.g. dieticians) Financial compensation for time spent (e.g. appropriate Medicare codes) [10] Models of care [61]

Finally, participants who take part in training report appreciating resources they can keep and refer back to [40] and resources they can give to women [9, 10, 45] to help them facilitate a discussion regarding weight gain. Well-liked resources include food logs [67] and reputable websites [9]. If there are other services such as a dietician clinic or weight management service, appropriate referral to those services should be included.

### Training Delivery

To date, gestational weight gain training has been delivered in a myriad of manners. This includes one-off face-to-face sessions [37, 43, 44], webinars and online training [37, 43] and training divided into several sessions [43, 46]. In the few studies that have compared delivery type, outcomes seem to be similar in terms of increased confidence [37, 43] with larger increases in knowledge for participants who attended face-to-face training [37]. Online learning is sometimes perceived negatively due to the perceived lack of interaction [6]; however, it can be used together with face-to-face sessions providing blended learning.

Common for many studies is a low attendance and completion rate regardless of delivery type [43], with some suggestion that completion rates are lower for online learning sessions [37]. These low rates may be due to lack of time. It is important, therefore, to provide training during work hours [43] and including training in mandatory education [6, 44, 50] or new starter orientation [44] which seems a promising strategy. Another barrier to attending training is other competing clinical priorities as identified by the individual healthcare professional or service management. Thus, even when training is part of mandatory education, there may be constant negotiation to keep gestational weight gain on the agenda, and local evidence may be needed to justify training [44]. In one study, a half-day training was negotiated between the training delivery team and their local maternity services [40].

Regardless of length and delivery of teaching, research suggests a number of activities that help participants engage with the topic and enhance their learning. These include quizzes to test ones learning, group discussion of case studies [43] and role play [6]. The latter two activities may be particularly useful for discussing how to speak about weight in a sensitive manner. It is also important to provide space for healthcare professionals to reflect on their training and practice [50, 51]. Training should be considered an ongoing process [46] with time to review learning and practice. This could be facilitated by, for example, listening back to conversations with women [46].

In summary, a blended learning approach using both face-to-face sessions and online resources may be a pragmatic way to deliver gestational weight gain training to time-poor healthcare professionals. Furthermore, with less time available for training, activities such as role play, discussion of case studies and reflection on practice may be good use of time. Incorporating education into undergraduate syllabi focusing on the *how* to discuss gestational weight gain may reduce the need for healthcare professional training in the future.

## Recommendations for Supporting Effective Conversations Regarding Weight Gain with Pregnant Women in Routine Antenatal Care

Training sessions that increase healthcare professionals’ knowledge and confidence to deliver conversations around gestational weight gain are unlikely to impact considerably on women’s weight gain on their own. There are system-level changes needed to facilitate and support these discussions. On a national level, these changes include gestational weight gain guidelines regarding recommended weight gain and routine weighing. Currently, national guidelines vary, and many countries do not have recommendations regarding weight gain [68]. A lack of national guidelines may erroneously suggest to healthcare professionals and pregnant women that gestational weight gain is not of clinical importance.

On a local level, time and appropriate equipment to weigh women is important. Routine weighing can normalise weighing and help instigate conversation regarding gestational weight gain [7]. A recent UK study found that a single 3-h training session was enough to prepare midwives to use antenatal weight charts, although this was not enough to influence women’s weight gain [69]. This was likely due to the midwives not being able to also incorporate the motivational interviewing techniques they learnt into clinical practice. These findings are in line with another study where midwives were trained to weigh women, plot their weight on a graph and provide brief feedback without any change in number of women gaining excessive gestational weight [70]. Clearly, more is needed to influence women’s gestational weight gain.

Findings from two interventions [41, 62, 63, 71] suggest that to have an effect on women’s weight gain, considerable practice changes are needed. In the Mighty Mums study, midwives received training, resources for themselves and the women, weighed women in longer than normal appointments and could refer women to other diet and physical activity services [62, 63]. Overall, there was no difference in the number of women gaining an excessive amount of weight; however, in the per-protocol group where women engaged in some of the intervention, the intervention group gained less weight compared to the control group. In a different study, Lindberg and colleagues implemented a ‘best practice alert’ into their electronic medical record where the woman’s weight gain was calculated and a conversation and related documentation was prompted regarding gestational weight gain and helped the healthcare professional refer to appropriate services such as gestational diabetes testing. Together, this led to better documentation regarding women’s weight [41] and more women met the IOM guidelines for gestational weight gain [71]. These findings are in line with other research where changes to the practice environment has improved documentation of weight [72••] and where changes within electronic medical records show most potential [73••].

Another local factor that will aid initiating a conversation about gestational weight gain is having a dietician or weight management service to refer to. This was part of the Mighty Mums intervention, and meeting with the dietician was associated with better weight outcomes [63]. When referring to an additional service, it is important that it is appropriate as some women may not be able to afford or have time to attend another service [9, 74]. Having other services available could also increase healthcare professionals’ confidence to initiate the discussion regarding gestational weight gain, as they can refer to others for further support [6]. Associated with this is the interpersonal factor of having an established relationship with the woman, ideally through a continuity of care model, which can support regular gestational weight gain discussions [3, 7, 61, 75].

Finally, it is important that women receive consistent information from all healthcare professionals they come in contact with during pregnancy [7]. This may include midwives, obstetricians, family physician/general practitioner, nutritionists or dieticians, exercise professionals, maternal-foetal medicine and other specialists [14]. More work is needed regarding the role of different healthcare professionals to ensure consistent care [6]. This should also include healthcare professionals that women meet postnatally such as maternal and child nurses so that women can continue to receive weight management support [76]. In other words, multidisciplinary training regarding gestational weight gain is needed [3, 10] and could start at pre-registration level [76].

## Future Directions

The amount of published research regarding training of healthcare professionals and implementation initiatives in the practice environment to support women gain a healthy weight in pregnancy has increased over the last 10 years. It is important that the multilevel factors (of which some are outlined in Table 1) influencing this discussion are acknowledged and targeted in future research. For example, what combination of resources and practice changes may be most effective at improving gestational weight gain support? This can include support from hospital administrators, new documentation, services and pathways and management support. Further, economic evaluations are needed to test intervention cost-effectiveness. In other words, there is still work to be done to identify best practice for healthcare professional outcomes and women’s outcomes [22••]. This review has identified several promising strategies to include in these interventions such as training development, content and delivery. However, future interventions need to go beyond healthcare professional training to measure the impact of the training on women and their behaviours and weight. Intervention impact is likely to depend on healthcare professional and women’s characteristics and type of relationship, and these should therefore be measured and long-term outcomes collected [43]. Future training should also aim to be theory-based and explicit regarding its content [77].

More work also needs to be done with women and their partners prior to conception, to make them aware of risks associated with excessive gestational weight gain. We know that preconception women have inaccurate beliefs and knowledge about gestational weight gain [16], making it even more important to discuss this in early pregnancy. Further, women asking about weight gain help and motivate healthcare professionals to discuss this issue [9, 19]. Herein lie opportunities to educate and train healthcare professionals who work with preconception women (e.g., family physicians) to include the importance of optimising pre-pregnancy weight and gestational weight gain in preconception counselling sessions. This is particularly important because evidence highlights that many women gain a significant amount of weight before their first antenatal visit and this early weight gain is associated with overall high gestational weight gain and adverse pregnancy outcomes [78].

## Strengths and Limitations

This is a narrative review and as such may not be considered as rigorous and robust as a systematic review. That said, using an existing systematic review as a starting point [22••], we then scoped the literature for training and interventions published through forward citations. This led us to identify recent evaluations of healthcare professional training but also qualitative and quantitative research and systematic reviews that have corroborated our findings and informed our recommendations. There is always a risk, as with all reviews, that some relevant research has been omitted.

## Conclusion

In conclusion, a number of evaluated healthcare professional training sessions show promising results in terms of increased participant confidence and knowledge and women’s outcomes. It is clear that the interventions that have also implemented resources in the practice environment are the ones most likely to influence gestational weight gain. More work is now needed to identify the most useful training resources and practice changes to support women gain a healthy weight in pregnancy.
